# MicroRNAs as Biomarkers and Therapeutic Targets in Doxorubicin-Induced Cardiomyopathy: A Review

**DOI:** 10.3389/fcvm.2021.740515

**Published:** 2021-11-24

**Authors:** Liuying Chen, Yizhou Xu

**Affiliations:** ^1^Zhejiang Chinese Medical University, Hangzhou, China; ^2^Department of Cardiology, Affiliated Hangzhou First People's Hospital, Zhejiang University School of Medicine, Hangzhou, China

**Keywords:** doxorubicin, microRNAs, cardiotoxicity, biomarkers, treatment strategy

## Abstract

Doxorubicin is a broad-spectrum chemotherapy drug applied in antitumor therapy. However, its clinical utility is limited by its fatal cardiotoxicity. Doxorubicin (DOX)-induced cardiomyopathy (DIC) begins with the first DOX dose and is characterized by being cumulative dose-dependent, and its early diagnosis using common detection methods is very difficult. Therefore, it is urgent to determine the underlying mechanism of DIC to construct treatment strategies for the early intervention before irreversible damage to the myocardium occurs. Growing evidence suggests that microRNAs (miRNAs) play regulatory roles in the cardiovascular system. miRNAs may be involved in DIC by acting through multiple pathways to induce cardiomyocyte injury. Recent studies have shown that the dysregulation of miRNA expression can aggravate the pathological process of DIC, including the induction of oxidative stress, apoptosis, ion channel dysfunction and microvascular dysfunction. Current findings on the roles of miRNAs in DIC have led to a wide range of studies exploring candidate miRNAs to be utilized as diagnostic biomarkers and potential therapeutic targets for DIC. In this review, we discuss frontier studies on the roles of miRNAs in DIC to better understand their functions, develop relevant applications in DIC, discuss possible reasons for the limitations of their use and speculate on innovative treatment strategies.

## Background

Doxorubicin (DOX) is an effective anthracycline agent used to treat a wide range of malignant tumors. However, its dose–response cardiotoxic side effects are known to lead to doxorubicin-induced cardiomyopathy (DIC), which severely limits the antitumor therapeutic utility of DOX (1, 2). Although several mechanisms behind the pathological changes in the heart were reported, such as DNA structural damage, RNA/protein synthesis inhibition and autophagy dysregulation, many important points still need investigation (3–5). On the one hand, there is a lack of distinctive biomarkers for early diagnosis of DIC. On the other hand, there is a need for further cardioprotective medications for DIC therapy other than the angiotensin-converting enzyme (ACE) inhibitors, which have limited benefits (6). Therefore, there is an urgent need to explore additional novel biotherapies for DIC and effective, non-invasive and highly specific biomarkers for its early identification.

With the rapid advancements in gene sequencing, several studies have investigated the microRNAs (miRNAs) in DIC. miRNAs are non-coding RNA molecules consisting of 19–25 nucleotides, which regulate the posttranscriptional silencing of target genes (7). A single miRNA may target hundreds of mRNAs and influence the expression of many genes, many of which are involved in a functional interacting pathway. Besides, miRNAs play critical roles in many cellular biological processes under different physiological and pathological conditions in cardiovascular diseases, including differentiation, replication and regeneration (8–10). Therefore, miRNAs represent attractive diagnostic and therapeutic targets in cardiovascular diseases. As the interest in the functions of different miRNAs is steadily increasing, questions about their true role in the DIC development and their potential as a therapeutic approach are constantly being raised.

## miRNAs in the Development of Doxorubicin-Induced Cardiomyopathy

miRNAs play an important role in regulating DIC progression by regulating multiple targets involved in oxidative stress, apoptosis, calcium homeostasis and vascular homeostasis. In the following sections, we summarize the specific roles of miRNAs in the primary aspects of DIC development. A list of reported miRNAs with their performance and targets in DIC development in animal models is presented in Table 1.

**Table 1:** miRNAs in DIC development in animal models.

**miRNAs**	**Model**	**Cumulative dose**	**Sample**	**Cardiotoxicity assessment**	**Regulation**	**Functions**	**Target**	**References**
miR-21	Mice	20 mg/kg	Heart	Echocardiography, cardiac catheterization, histological assessment, biochemical detection	Up	Positive	B cell translocation gene 2	(11)
miR-22	Mice	16 mg/kg	Heart Cardiomyocytes	Echocardiography, histological assessment, biochemical detection	Up	Negative,	Sirt1	(12)
miR-25	Mice	20 mg/kg	Heart	Echocardiography, histological assessment	Up	Negative	PTEN	(13)
miR-34a	Mice	12 mg/kg	Heart Plasma	Troponin T (TnT)	Up	Unreported	Unreported	(14)
miR-124	Mice	12 mg/kg	Heart	Biochemical detection	Down	Positive	p66Shc	(15)
miR-31-5p	Mice	20 mg/kg	Heart	Echocardiography, histological assessment	Up	Negative	Quaking and circPan3	(16)
miR-133b	Mice	4 mg/kg/once for 16 times	Heart	Echocardiography, histological assessment	Down	Positive	PTBP1 and TAGLN2	(17)
miR-140-5p	Mice, rats	15 mg/kg/d for 8 days	Heart	Echocardiography, electrocardiogram, histological assessment, biochemical detection	Up	Negative	Nrf2 and Sirt2	(18)
miR-143	Mice	15 mg/kg/d for 8 days	Heart	Echocardiography, cardiac catheterization, histological assessment, biochemical detection	Up	Negative	AKT	(19)
miR-146	Mice	30 mg/kg	Heart	Echocardiography, histological assessment	Expression showed a concentration-dependent and time-dependent increase, and decreased to a certain extent	Positive	TAF9b/P53 pathway	(20)
miR-146a	Mice	20 mg/kg	Cardiomyocytes	NA	Upregulation	Negative	ErbB pathway	(21)
miR-152	Mice	15 mg/kg	Heart Cardiomyocytes	Echocardiography, cardiac catheterization, biochemical detection	Down	Positive	Nrf2	(22)
miR-200a	Mice	20 mg/kg	Heart Cardiomyocytes	Echocardiography, cardiac catheterization, biochemical detection	Down	Positive	Nrf2	(23)
miR-204	Mice	15 mg/kg	Heart	Echocardiography, hemodynamic analysis, histological assessment, biochemical detection	Down	Positive	HMGB1	(24)
miR-208a	Mice	20 mg/kg	Heart	Echocardiography	Down	Negative	GATA4	(25)
miR-212/132 Cluster	Mice	25 mg/kg	Heart	Echocardiography, histological assessment	Up	Positive	*Fitm*2	(26)
miR-320a	Mice	25 mg/kg	Heart	Echocardiography, hemodynamic analysis, histological assessment, biochemical detection	Up	Negative	VEGF signal pathway	(27)
miR-375	Mice	15 mg/kg/d for 8 days	Heart Cardiomyocytes	Echocardiography, cardiac catheterization, biochemical detection	Up	Negative	PDK1/AKT pathway	(28)
miR-526b-3p	Mice	25 mg/kg	Heart	Histological assessment	Up	Negative	STAT3/VEGFA pathway	(29)
let-7g	Rats	6, 12 and 18 mg/kg	Heart	Heart rate, blood pressure, cardiac troponin T	Down	Unreported	Unreported	(30)
miR-30	Rats	15 mg/kg	Heart Cardiomyocytes	NA	Down	Positive	β-adrenergic signaling	(31)
miR-30e	Rats	10 mg/kg	Heart	Echocardiography	Down	Positive	Beclin-1	(32)
miR-34a-5p	Rats	16 mg/kg or 32 mg/kg	Heart Plasma	Echocardiography, histological assessment, biochemical detection	Up	Negative	Sirt1/p66shc pathway	(33)
miR-378	Rats	8 mg/kg	Heart cardiomyocytes	Echocardiography, histological assessment	Down	Positive	Calumenin	(34)

## miRNAs in Doxorubicin-Induced Oxidative Stress

Although DOX causes cardiomyocyte damage through multiple targets, one of the most important mechanisms of DIC is mitochondrial dysfunction is characterized by inhibited mitochondrial respiration (35). DOX is a favorable substrate for reduction by a number of oxidoreductases within the cell, including NADPH-dependent cytochrome P450 reductase, NADH-dehydrogenase of mitochondrial complex I, and assorted soluble oxidoreductases present in the cytoplasm (36). DOX redox cycles primarily on the NADH-dehydrogenase of mitochondrial complex I of the mitochondrial respiratory chain reduces the DOX to form a highly reactive semiquinone, which in turn results in increasing the intracellular reactive oxygen species (ROS) production and oxidative stress after reacting with oxygen (1, 37, 38). The increased production of ROS results from the electron exchange between the anthracycline quinone moiety and oxygen molecules and other cellular electron donors, as well as the DOX-iron complexes that undergo redox cycling and generate oxygen radicals (39, 40).

When it comes to oxidative stress, miRNAs are strongly linked to a balance in it. miR-22 is a cardiac-enriched microRNA, which functions as a key regulator in DOX-induced cardiac injury by directly binding to the 30-UTR of Sirtuin-1 (SIRT1), an NAD+-dependent deacetylase, causing Sirt1 downregulation (12). This regulation suppresses the protection mediated by SIRT1, which is highly sensitive to cellular redox states and can counteract the ROS effects through the deacetylation of multiple cellular targets to confer cardioprotection and maintenance of the vascular function (41, 42). Conversely, the inhibition of miR-22 upregulates the expression of SIRT1 and enhances its beneficial effects (12). Similarly, the miR-34 family and miR-140-5p are also involved in partly suppressing SIRTs to aggravate the doxorubicin-induced cytotoxicity (18, 33, 43). Further studies have shown that the overexpression of miR-34a-5p inhibits SIRT1 to upregulate p66Shc, an isoform of the shcA adapter molecule that is involved in promoting the development of DIC by modulating intracellular redox balance; it achieves this by increasing the ROS concentration, thus acting as a critical mediator of intracellular oxidative signal transduction (33, 44). miR-25 has also been reported to increase the ROS production. The overexpression of miR-25 accelerates DNA damage by negatively controlling the expression of phosphatase and tensin homolog deleted on chromosome ten (PTEN), which further aggravates the doxorubicin-induced PTEN suppression, suggesting the alleviation of oxidative stress (13, 45). Previous studies indicated that AKT signaling pathway is involved in ROS-mediated myocardial injury. The activation of AKT signaling pathway exerts the suppressive effect on intracellular ROS production and antioxidative enzyme reduction (46). However, AKT is deactivated in response to DOX administration in the heart tissue, while the activation of AKT promotes the vitality of cardiomyocytes and reduces DOX-induced cardiac oxidative stress (47, 48). This effect has been shown to be exerted by DOX through increasing the expression of miR-143 and miR-375 (19, 28).

In contrast, some miRNAs that exert beneficial effects represent attractive therapeutic targets in DIC progression. For example, miRNAs upregulate the nuclear factor (erythroid-derived 2)-like 2 (Nrf2), which encodes multiple antioxidant genes and detoxifying enzymes to counteract oxidative stress, serving as an intracellular defense mechanism to attenuate DIC (49). Evidence has demonstrated that the overexpression of miR-152 in DOX-induced cardiotoxicity activates the Nrf2 signaling pathway to inhibit DOX-related ROS and superoxide (22). miR-200a has been shown to play a similar role in attenuating DIC by upregulating Nrf2 *in vivo* (23). In addition, unlike miR-34a-5p, the overexpression of miR-124 downregulates the expression of p66Shc to inhibit p66Shc-mediated oxidative stress, decrease the activity of malondialdehyde (MDA) and increase the activity of superoxide dismutase (SOD) (15). Interestingly, the above-mentioned miR-140-5p, which is a multitarget RNA, also decreases the protein levels of Nrf2 in addition to SIRT2 (18).

These findings demonstrate that miRNAs participate in oxidative stress in DIC by providing a multipathway of molecular regulation in cardiomyocytes, which demonstrates the significance of miRNAs in DIC development.

## miRNAs in Doxorubicin-Induced Apoptosis

Cardiomyocyte apoptosis is usually accompanied by excessive oxidative stress, which involves the adaptive and programmed death of cardiomyocytes in DIC. Many mechanisms behind the effects of miRNAs on DOX-induced cardiomyocyte apoptosis have been reported.

miRNAs are effective in accelerating the progression of DIC by controlling the transcription levels to mediate DOX-induced apoptosis of cardiomyocytes (50). For example, miR-21 was one of the first miRNAs identified in mammals, and its activation is present in many types of tumors as well as cardiovascular diseases, such as myocardial infarction and fibrosis (51, 52). It has been reported that the expression of miR-21 expression modestly increases in the cardiomyocytes of chronically treated DOX models. According to several experiments, the bioinformatics-based prediction of miR-21 targets demonstrated that the B cell translocation gene 2 (BTG2) is involved in the regulation of miR-21 chronic DOX-induced injury (11). BTG2 has been investigated for its functions in cell proliferation, cycle regulation and apoptosis, especially in tumor cells (53).

Another effect mechanism of miRNAs on DOX-induced apoptosis of cardiomyocytes is exacerbating it by participating in the regulation of circular RNAs (circRNAs). For example, miR-31-5p controls Quaking (QKI), an RNA-binding protein (RBP) that has been reported to have a key function in multiple RNA biological functions, ultimately regulating the formation of circRNA in DIC (54). Moreover, the overexpression of QKI significantly alleviates apoptotic cell death, while silencing it downregulates the maturation of circRNAs derived from Pan3 (circPan3) in cardiomyocytes, which exacerbates the cardiotoxicity of DOX (16).

Dox-induced cardiomyocyte apoptosis also results from the depletion of cardiac enriched transcription factors, such as GATAs, which control the function of miRNAs in DIC (25, 55). For example, the NRG-1/ErbB signaling pathway has been long proposed to play an important role in heart development (56). The overexpression of miR-164a induces the suppression of the neuregulin-1-ErbB pathway, which aggravates acute doxorubicin cardiotoxicity; this was achieved through targeting the ErbB4 3′ UTR to enhance the DOX-induced apoptosis (21). Interestingly, another study showed that miR-146a attenuated apoptosis in DOX-induced cardiotoxicity. We observed that its expression significantly depended on the time and dose of DOX, which was not mentioned in other studies (20). This finding suggests that the role of miRNAs in DIC may change with the cumulative dose of DOX rather than being immutable.

Current studies also miRNAs could regulate apoptosis by binding to RNA-binding proteins. For example, the polypyrimidine tract binding protein 1 (PTBP1), which plays a role in the functional organization of the actin filament, and transgelin 2 (TAGLN2), which is likely anti-apoptotic, could bind to miR-133b (17, 57, 58). Moreover, miR-133b negatively regulates the protein expression levels of PTBP1 and TAGLN2, while the overexpression of PTBP1 or TAGLN2 reverses the effects of miR-133b on apoptosis and collagen accumulation. These results indicate that miR-133b may alleviate DOX-induced cardiomyocyte apoptosis and cardiac fibrosis by targeting PTBP1 and TAGLN2.

Finally, although numerous studies focusing on the function of a single miRNA in DIC have been performed, the development of DIC may not be mediated by a single RNA. Studies have demonstrated that DIC is the cooperative effect of multiple RNAs. For example, the overexpression of the miR-212/132 cluster inhibits DOX-induced atrophy, and its downstream mediator is Fitm2, which has miR-212/132 binding sites in its 3′UTR (26). However, the miR-212/132 cluster was initially found to induce cardiomyocyte hypertrophy. Hence, the mechanism of the miR-212/132 family in regulating the different functions of cardiomyocyte morphology is still unclear (59, 60).

## miRNAs in Doxorubicin-Induced Disruption of Calcium Homeostasis

Calcium homeostasis represents one of the most important balanced systems in cardiomyocytes, which plays a crucial role in maintaining mitochondrial function and excitation-contraction coupling, thus contributing to the myocardial electrical activity and contractile function (61). Under the stimulation of DOX, the expression of sarcoplasmic/endoplasmic reticulum Ca2^+^ ATPase2a (SERCA2a) is down-regulated (62, 63). This Ca2^+^ pump responsible for Ca2^+^ influx from cytosol to sarcoplasmic reticulum, whose dysfunction elevates both cytosolic and mitochondrial Ca2^+^ levels, resulting in activation of contractile proteins and initiation of muscle contraction (62, 64). Moreover, whenever the mitochondrial Ca2^+^level reaches a certain threshold, the opening of a mitochondrial permeability transition pore is triggered, cytochrome-C then is allowed to release into the cytosol and hence formation of autophagosome, which in turn activates caspase-9 and ultimately result in apoptotic cell death (65). It can be seen that the disturbed calcium homeostasis is also an important part of DOX-induced cardiotoxicity.

Calumenin is a calcium-binding protein that regulates calcium homeostasis in mammalian cardiomyocytes (66). miR-378^*^ was reported to decrease the expression of calumenin H9C2 cells (67). In DIC, the overexpression of miR-378^*^ upregulates the expression of calumenin to inhibit DOX-induced ER stress and attenuate DOX-induced myocardial apoptosis, while the specific mechanism is not yet clear (34).

Meanwhile, the relationship between miR-133a and calcium homeostasis has been investigated in a DOX-induced cardiotoxicity model. Although a modest increase in miR-133a was observed, the downstream effectors of miR-133a in DOX-treated hearts were not statistically significant (68). Moreover, calcium homeostasis is very likely to be affected by genetic factors and acquired diseases. Studies have pointed out that the intracellular calcium overload caused by hypoxia is regulated by miR-19a by targeting Na (+)/H (+) exchanger 1 in coronary microvascular obstruction injury (69).

## miRNAs in Doxorubicin-Induced Abnormal Vascular Homeostasis

DOX-induced cardiotoxicity does not only accumulate in myocardial cells but also targets the endothelial cells of the small blood vessels in the heart tissue. Usually, the endothelial cells form a physical barrier to protect cardiomyocytes from circulating toxic substances and secrete cardioprotective molecules, including nitric oxide, endothelial-derived prostaglandin I2, and proteins such as neuregulin-1 and endothelin-1 that support cardiomyocytes functions (70). However, by destructing the endothelial cell dysfunction, DOX will make cardiomyocytes more susceptible to be damaged (71). Dox-induced abnormal vascular homeostasis is a pathological event that is critical to cardiovascular complications, and its damage to the heart is no less than that of DOX, impairing the cardiomyocyte itself (72). Vascular endothelial growth factor (VEGF) is thought to be one of the most important factors regulating the homeostasis of the vascular endothelium. There is currently considerable evidence demonstrating that miRNAs are involved in the regulation of endothelial cell function through the VEGF pathway and are closely related to cardiovascular diseases (73).

For example, miR-320a plays a negative role in the vascular endothelial function, since its level is upregulated by DOX, while the cardiac microvessel density is relatively decreased (27). Moreover, the knockdown of miR-320a exerts cardioprotection by enhancing the proliferation and inhibiting the apoptosis in cultured endothelial cells to attenuate the DOX-induced cardiac pathology. This finding further verified the function of VEGF-A in doxorubicin impairment in the human umbilical vein endothelial cells (HUVECs). siRNA against VEGF-A transfected into HUVECs led to exaggerated DOX-induced damage, illustrating that it was the VEGF signal pathway targeted by miR-320a after DOX treatment, but the regulatory mechanism remains unknown.

In contrast, miR-526b-3p has been shown to aggravate DOX-induced cardiac abnormalities by regulating its transcription instead of directly binding its 3′UTR to inactivate VEGF-A (29). Bioinformatics analysis revealed two potent binding positions (BS1 and BS2) in the VEGF-A promoter for STAT3, which is a well-documented transcriptional activator. Hence, it was hypothesized that miR-526b-3p mediates the downregulation of VEGF-A by targeting STAT3.

These findings fully demonstrate the importance of miRNAs in abnormal vascular homeostasis caused by DOX and deserve to be explored in depth.

## miRNAs for the Diagnosis and Prognosis of Doxorubicin-Induced Cardiomyopathy

Most DIC patients are diagnosed at the terminal stage, due to the lack of precise indicators for the early diagnosis of DIC. Current DIC diagnosis depends entirely on routine cardiac function testing methods that have low specificity for DIC, such as echocardiography (37, 74). Therefore, there is a need for novel biomarkers with high sensitivity and specificity for DIC diagnosis at an early stage, especially for high-dose and long-term DOX-treated patients. miRNAs in serum can be easily obtained and detected using miRNA sequencing methods (10, 75, 76). Therefore, miRNAs have been investigated as non-invasive biomarkers for the early diagnosis of DIC (77). Table 2 summarizes the clinical significance of miRNAs and their potential roles in DIC.

**Table 2:** Clinical significance of miRNAs in DIC.

**miRNAs**	**Model**	**Sample**	**miRNAs selection**	**Expression in DIC**	**Potential role**	**References**
miR-1	Breast cancer patients	Serum	Screening	Upregulation	Arrhythmia, myocardial infarction, cardiac hypertrophy and heart failure	(78)
	Breast cancer patients	Serum	Screening	Downregulation		(79)
	Breast cancer patients	Serum	Screening	Unreported		(80)
	Cancer patients aged <18 years	Serum	Screening	Upregulation		(81)
miR-15a/b-5p	Breast cancer patients	Serum	Screening	Upregulation	Diffuse myocardial fibrosis, arteriogenesis and angiogenesis	(79)
miR-16-2-3p	Breast cancer patients	Serum	Screening	Upregulation	Sympathetic denervation and PPARγ activation	(79)
miR-16-5-3p	Breast cancer patients	Serum	Screening	Upregulation	Cardiac insufficiency	(79)
miR-29b,	Cancer patients aged <18 years	Serum	Screening	Upregulation	ECM remodeling	(81)
miR-23a-3p	Breast cancer patients	Serum	Screening	Upregulation	Cardiac insufficiency	(79)
miR-25a-3p	Breast cancer patients	Serum	Screening	Upregulation	Myocardial Infarction	(79)
miR-28a-3p	Breast cancer patients	Serum	Screening	Upregulation	Type 2 diabetes mellitus	(79)
miR-30d-5p	Breast cancer patients	Serum	Screening	Upregulation	Myocardial infarction	(79)
miR-34a-5p	Breast cancer patients	Serum	Screening	Upregulation	Cardiomyocyte apoptosis	(79)
	Diffuse large B-cell lymphoma patients	Serum	Screening	Upregulation		(33)
	Breast cancer patients	Serum	Screening	Unreported		(80)
miR-92a	Breast cancer patients	Serum	Screening	Upregulation	Pro-angiogenic	(79)
miR-122-5p	Breast cancer patients	Serum	Screening	Upregulation	Coronary artery disease	(80)
miR-133a-3p	Breast cancer patients	Serum	Screening	Downregulation	Proliferation, differentiation, survival, hypertrophic growth	(79)
miR-133b	Breast cancer patients	Serum	Screening	Downregulation	Proliferation, differentiation, survival, hypertrophic growth	(79)
	Breast cancer patients	Serum	Screening	Upregulation		(78)
miR-140-3p	Breast cancer patients	Serum	Screening	Upregulation	Myocardial infarction	(79)
miR-142-5p	Breast cancer patients	Serum	Screening	Upregulation	Inflammation, oxidative stress and apoptosis	(79)
miR-144-5p	Breast cancer patients	Serum	Screening	Upregulation	Proliferation, migration, invasion and apoptosis of human umbilical vein endothelial cells; identified in heart tissue	(79)
miR-145-5p	Breast cancer patients	Serum	Screening	Upregulation	Inflammatory response, apoptosis in cardiomyocytes	(79)
miR-205-5p	Breast cancer patients	Serum	Screening	Upregulation	Differentiation, capture and proliferation of breast cancer	(79)
miR-320a	Acute myelogenous leukemia	Serum	Screening	Downregulation	Endothelial injury	(27)
miR-324-5p	Breast cancer patients	Serum	Screening	Upregulation	Mitochondrial fission, apoptosis and myocardial infarction	(79)
miR-331-3p	Breast cancer patients	Serum	Screening	Upregulation	Tumor suppression	(79)
miR-363-3p	Breast cancer patients	Serum	Screening	Upregulation	Tumor suppression	(79)
miR-376a-3p	Breast cancer patients	Serum	Screening	Downregulation	Tumor suppression	(79)
miR-421	Breast cancer patients	Serum	Screening	Upregulation	Cardiomyocyte apoptosis, myocardial infarction	(79)
miR-486-5p	Breast cancer patients	Serum	Screening	Upregulation	Protection against cardiomyocyte apoptosis	(79)
miR-499	Cancer patients aged <18 years	Serum	Screening	Upregulation	Reactive oxygen species and mitochondrial dysfunction within the cardiomyocyte	(81)
miR-499a-5p	Breast cancer patients	Serum	Screening	Upregulation	Acute myocardial infarction	(80)
miR-501-3p	Breast cancer patients	Serum	Screening	Upregulation	Alzheimer's disease, metastasis and hepatocellular carcinoma	(79)
miR-502-3p	Breast cancer patients	Serum	Screening	Upregulation	Triple negative breast cancer	(79)
miR-532-3p	Breast cancer patients	Serum	Screening	Upregulation	Mitochondrial fission, apoptosis in the presence of doxorubicin	(79)
miR-532-5p	Breast cancer patients	Serum	Screening	Upregulation	Acute myocardial infarction, apoptotic	(79)
miR-660-5p	Breast cancer patients	Serum	Screening	Upregulation	Platelets, thrombotic events and acute myocardial infarction	(79)
miR-885-5p	Breast cancer patients	Serum	Screening	Upregulation	Liver toxicity	(80)
miR-1260a	Breast cancer patients	Serum	Screening	Upregulation	Metastatic melanoma	(79)
miR-1273g-3p	Breast cancer patients	Serum	Literature screening	Upregulation	Cell-cell adhesion, response to toxic substance, and lipid catabolic process	(82)
miR-4638-3p	Breast cancer patients	Serum	Literature screening	Downregulation	Cell-cell adhesion, response to toxic substance, and lipid catabolic process	(82)

Most of the studies on circulating miRNA profiles in DIC have focused on breast cancer patients treated with doxorubicin (78–80). Most miRNA were upregulated, while only a few miRNAs were downregulated in response to DOX treatment, such as miR-320a, miR-376a-3p and miR-4638-3p. The muscle-specific microRNA of miR-1 is the most abundant miRNA in the adult mouse heart, representing up to 40% of all miRNA transcripts (83). It has been widely studied due to its controversial level of expression.

A previous study revealed that breast cancer patients who received DOX treatment with elevated levels of cardiac troponin I (cTnI) exhibited higher circulating miR-1 levels than those who did not (78), while the levels of miR-133b,−146a,−208a/b and−423-5p did not show any significant difference in response to DOX treatment. These data demonstrate that only miR-1 has more sensitivity than whole serum in DIC, which makes it serum levels a novel biomarker for DIC. Furthermore, the results of the receiver operator characteristic (ROC) analysis indicated that miR-1 exerts a superior ability compared with cTnI for distinguishing cardiotoxicity (78). A similar performance of miR-1 was also confirmed in another study on DOX cardiotoxicity in cancer patients aged <18 years (81). However, a study screening circulating miRNA profiles of DOX-induced cardiotoxicity in breast cancer patients obtained the opposite result. This study used the left ventricle ejection fraction (LVEF) to evaluate the cardiac function. After miRNA expression detection, the data finally revealed downregulation of miR-1 (79).

Numerous studies have indicated the dysregulation of the miR-1 expression in cardiovascular diseases. miR-1 expression is decreased in chronic cardiac hypertrophy or heart failure and coordinated ventricular remodeling, while it is overexpressed in cardiac arrhythmias and depolarizes the cytoplasmic membrane (84–86). The elevated serum levels of miR-1 within 3 h of the onset of acute chest pain have been shown to have important clinical significance in the diagnosis and prognosis of acute myocardial infarction (87). These findings indicate that the expression of miR-1 usually changes without a fixed pattern and is always affected by the type and stage of the disease. Therefore, the sequential changes of miRNA-1 in a certain disease are of interest. Meanwhile, the differences in miRNA-1 expression of multiple cardiovascular disease spectrum in a fixed time and their mechanisms are also worthy of in-depth study.

In general, miRNAs like miRNA-1 have marked effects on the diagnosis and prognosis of DIC as well as predicting the progression of DOX-induced cardiotoxicity. However, different studies have obtained contradicting conclusions, even regarding the same miRNA. The absence of fixed principles to distinguish the roles of miRNAs in DIC implies that changes in miRNAs levels should be carefully observed. Furthermore, basic factors, such as the age, sex, complications and medication history of the patients should be considered when analyzing the differential expression of miRNAs. It is foreseeable that an increasing amount of miRNA biological information will be developed with the continued improvement of RNA sequencing methods.

## Applications of miRNAs in Doxorubicin-Induced Cardiomyopathy Therapeutic Strategies

The role of miRNAs in the treatment of DIC has been investigated, and many recent studies have shown that miRNAs represent an attractive target for DIC therapy. On the one hand, all the above-mentioned miRNAs can be used as indicators for DIC risk stratification and the evaluation of efficacy during treatment. More importantly, researchers can use specific miRNAs as targets for precision medicine or drug development. Hence, miRNAs and their upstream or downstream factor inhibitors or activators can be used as a potentially useful tool against DIC when these miRNAs are themselves pathogenic RNAs or key links to pathology.

One method is to transport cardioprotective miRNAs directly to the DIC heart. For example, a previous study tested the effects of inhibiting miR-375 in DIC mice (28). It demonstrated that a single subcutaneous injection of miR-375 inhibitor downregulated the upregulated miR-375 in DIC, and then restored the redox homeostasis of cardiomyocytes. Meanwhile, exogenous supplementation of beneficial RNA after DOX treatment also represents a novel treatment. miR-21 is a protective myocardial RNA, and delivering protective miR-21 holds promise for cardiotoxicity prevention and therapy. The Sun group has innovatively delivered miR-21-loaded exosomes into the circulation to treat DIC mice using ultrasound-targeted microbubble destruction (UTMD) (88). First, miRNA mimics were loaded into exosomes, a delivery carrier isolated from the plasma, and then the combination was injected into the circulation (89). Next, UTMD was utilized to promote miR-21 delivery into the heart. Strikingly, cell death in the heart was significantly decreased, while the cardiac function in a DOX-induced cardiotoxicity mouse model was restored.

In addition, we can also choose the drug as the object under miRNA guidance. Many existing drugs are currently being tested with mechanisms that interfere with miRNA dysregulation or downstream to cure DIC. For example, dexrazoxane, which is a cardioprotective agent, has been examined in many clinical trials, but its ability to protect cardiomyocytes from DOX-induced apoptosis by modulating miR-17-5p has only been tapped (90, 91). Furthermore, many new drugs are being investigated. Great progress has been made for compounds derived from natural products. For example, paeoniflorin (PEF) is a monoterpene glucoside derived from *Paeonia lactiflora* Pall. PEF has been confirmed to upregulate the expression of miR-1, concomitantly with markedly decreased ROS generation, further relieving cardiomyocyte apoptosis (92). Another example is apigenin, in common plants, fruits and vegetables. Apigenin contains a bioactive flavonoid that has anti-inflammatory, anti-apoptotic, antioxidant and anticarcinogenic properties, which has indeed relieved oxidative stress in a DOX model (93).

Another domain where miRNAs can be used to suppress DIC-development is miRNA-related cell-based therapy. Cell-based therapy has huge potential for treating and even curing cardiovascular diseases. Bone marrow-derived mesenchymal stem cells (MSCs) are most commonly reported due to their regenerative properties and known biosafety (94), and its resistance to DOX-induced cardiac senescence has been intensively studied. The Xia group used the Transwell system to coculture H9c2 cells and MSCs (95). MSCs exerted an inhibitory effect on the senescence-related genes of p53 and p16 in response to DOX intervention. This study also revealed that MSCs rescued DOX-induced senescence by inhibiting the miR-34a-SIRT1 axis. However, this experiment was only verified in H9c2 cells, and the effect of the overall models needs to be verified.

Overall, the above-mentioned findings have shown that miRNAs have large potential to be exploited in suppressing DIC development. miRNAs present attractive opportunities to guide novel strategies to treat DIC. Unfortunately, there are still some limitations and challenges in the study of applying miRNAs. Firstly, the most common feature among these studies was heart dysfunction that was verified only by instrumental evaluation (e.g., echography) or by the dosage of circulating markers (e.g., troponins) at a single time point; this ignores the relationship between the dose of DOX and heart injury, leading to an inaccurate control of time for cardiotoxicity. However, the regulation of miRNAs is closely related to the dose of DOX and duration of the disease. Therefore, assessing the correlation between the expression of miRNAs and DOX accumulation without a dynamic dose-effect evaluation resulted in an incomplete description of the real role of miRNAs in DIC and reducing the specificity and sensitivity of miRNA for diagnosing DIC. Secondly, there is a lack of miRNA matching between heart samples and blood samples to jointly confirm the changes of these miRNAs. miRNA expression in human circulation samples lack confirmation or comparison with heart samples. Indeed, human-based studies pose greater challenges than animal models due to the difficulty in acquiring heart tissue samples. As a result, this makes the estimation of the location of miRNA expression difficult. Moreover, in the process of clinical application, the economic cost and time cost of miRNA detection should also take into consideration. Exploring effective methods of miRNA-mediated DIC treatment is a prospective direction that requires additional effort.

## Conclusions

Doxorubicin-induced cardiomyopathy is a public health concern that has received increasing attention due to the high incidence of malignant tumors and widespread use of anthracycline agents (96). In the past few years, the role of miRNAs in the process of DOX-induced cardiotoxicity has been approached, and many treatment strategies have been formulated to target miRNAs. In conclusion, this review summarized and updated the recent advances regarding the functions of miRNAs in DIC development and their clinical significance as well as application potential. However, we acknowledge that many gaps in this research field still need to be filled. Exploiting the potential of miRNAs as new therapeutic modalities may prove to be helpful for DIC patients in the future.

## Author Contributions

LC conceived the study and its design and drafted the manuscript. YX reviewed the manuscript. All authors contributed to the article and approved the submitted version.

## Funding

This work was supported by the Hangzhou Medical and Health Technology Project (No. Z20200135).

## Conflict of Interest

The authors declare that the research was conducted in the absence of any commercial or financial relationships that could be construed as a potential conflict of interest.

## Publisher's Note

All claims expressed in this article are solely those of the authors and do not necessarily represent those of their affiliated organizations, or those of the publisher, the editors and the reviewers. Any product that may be evaluated in this article, or claim that may be made by its manufacturer, is not guaranteed or endorsed by the publisher.
